# The C-type lectin receptor Dectin-2 is crucial for the development of experimental autoimmune skin disease

**DOI:** 10.3389/fimmu.2026.1962664

**Published:** 2026-10-28

**Authors:** Lili Balogh, Eszter Káposztás, Tamara Józsa, Viktor Gyula Kovács, Silvia Hayer, Stephan Blüml, Attila Mócsai, Tamás Németh

**Affiliations:** 1 Department of Physiology, Semmelweis University School of Medicine, Budapest, Hungary; 2 MTA-SE “Lendület” Translational Rheumatology Research Group, Hungarian Academy of Sciences and Semmelweis University, Budapest, Hungary; 3 Department of Internal Medicine III, Division of Rheumatology, Medical University of Vienna, Vienna, Austria; 4 Ludwig Boltzmann Institute for Arthritis and Rehabilitation, Vienna, Austria; 5 HUN-REN-SE Inflammation Physiology Research Group, HUN-REN Hungarian Research Network and Semmelweis University, Budapest, Hungary; 6 MTA-SE “Lendület” Inflammation Physiology Research Group, Hungarian Academy of Sciences and Semmelweis University, Budapest, Hungary; 7 Department of Rheumatology and Immunology, Semmelweis University, Budapest, Hungary

**Keywords:** Arthus reaction, epidermolysis bullosa acquisita, immune complex, inflammation, neutrophil

## Abstract

Autoimmune blistering skin diseases have deleterious effects on patients’ lives with a great (and unmet) need for more treatment options and potential new drug targets. C-type lectin receptors like Dectin-2 are pattern recognition receptors that bind to carbohydrate moieties on microorganisms leading to the elimination of the pathogens. Recently, Dectin-2 gene variants have been linked to autoimmune diseases, its expression in neutrophils has been shown to be upregulated in the autoimmune blistering skin disease epidermolysis bullosa acquisita (EBA), while we found that immune complex-stimulated neutrophils upregulated the gene of Dectin-2. These results prompted us to test the role of Dectin-2 in neutrophil- and autoantibody-mediated experimental inflammation. Dectin-2-deficient mice showed a nearly complete protection against the development of EBA, which was further validated in the reverse passive Arthus reaction. Immune cell accumulation and proinflammatory mediator levels were also decreased in Dectin-2^–/–^ mice. In line with these findings, postmigratory Fc receptor-dependent neutrophil, but not macrophage functions were dramatically reduced in the absence of Dectin-2. Our results show an essential role for Dectin-2 in immune complex-mediated *in vitro* neutrophil processes, in experimental EBA and immune complex-mediated inflammation, which raise the possibility that this C-type lectin receptor could be a treatment target in these disorders.

## Introduction

Subepidermal blistering skin diseases – like bullous pemphigoid (BP) or epidermolysis bullosa acquisita (EBA) – are chronic autoimmune disorders, where the epidermis and dermis are separated causing blisters and erosions, mainly due to immune cell activation triggered by the identification of the molecules of the dermal-epidermal junction (DEJ) as foreign components (1, 2). BP has a growing incidence, it mainly affects older individuals and while several therapeutic options are available, there are many challenges remaining for an optimal management of the disease in many patients (1). EBA is a relatively rare disorder, which is characterized by autoantibodies against the anchoring protein type VII collagen (CVII) (2). Based on the lack of randomized clinical trials, the therapy of EBA is far from well-established, meaning that there is a great (and unmet) need to introduce novel therapeutic agents in the management of the disease.

Animal models are considered to be important tools to better understand the pathogenesis of subepidermal blistering skin diseases and to identify new therapeutic targets. The passive transfer of anti-type VII collagen (anti-CVII) antibodies to mice can trigger skin disease resembling to the inflammatory form of human EBA, generating an *in vivo* system, which is one of the best working autoimmune blistering skin disease models (3, 4). The injected antibodies deposit in the dermal-epidermal junction and trigger inflammation by the help of complement and Fcγ receptor activation, causing immune cell (e.g. neutrophil) recruitment and dermal-epidermal separation leading to blisters, erosions and ulcers, which are classical hallmarks of the disorder (3, 5–7).

Dectin-2 is an Fc receptor γ-chain-associated, Syk-tyrosine kinase- and CARD9-coupled C-type lectin receptor, which is an important component of antifungal immunity (8, 9). We have previously shown that the Syk tyrosine kinase and the gene regulator adaptor protein CARD9 have essential roles in the myeloid compartment and especially in neutrophils in the development and progression of experimental EBA (10–12). C-type lectin receptors may be able to recognize the glycosylated forms of autoantibody Fc portions and Dectin-2 may also bind to carbohydrates like glycan mannose (e.g. on β-glucuronidase) in tissues, while participating in sterile inflammation (13, 14). In line with this, some Dectin-2 single nucleotide polymorphisms have been found to be associated with the susceptibility to rheumatoid arthritis, a systemic autoimmune disease (15). Furthermore, the Dectin-2 encoding *Clec4n* gene was found to be upregulated in neutrophils in the lesional skin in experimental EBA (16). In addition, in our hands, *Clec4n* showed a very strong upregulation in mouse neutrophils upon immune complex activation, pointing to a potential role for Fcγ receptors in the regulation of Dectin-2 expression in these granulocytes (12).

The above mentioned results prompted us to test the role of Dectin-2 in the neutrophil- and Fc receptor-dependent experimental EBA model. We observed a nearly total protection of Dectin-2-deficient mice from the development of experimental EBA. In addition, a similar *in vivo* phenotype was seen in the immune complex-dependent reverse passive Arthus reaction in the absence of Dectin-2. In line with these observations, immune complex-activated Dectin-2-deficient neutrophils showed decreased *in vitro* superoxide release, cell spreading and proinflammatory mediator production compared to wild type granulocytes, while their integrin-mediated migration was unaffected, pointing to altered postmigratory (immune complex-dependent) functions in the absence of the molecule. Our results raise the possibility that Dectin-2 could be a therapeutic target in the treatment of autoimmune blistering skin (and potentially some other immune complex-mediated) diseases in the future.

## Materials and methods

### Animals

Wild type C57BL/6J and C57BL/6N mice were purchased from the Hungarian National Institute of Oncology or were obtained from the Institute of Translational Medicine at Semmelweis University. Dectin-2-deficient C57BL/6 mice (referred to as Dectin-2^–/–^ animals) were obtained from Prof. Yoichiro Iwakura (Tokyo University of Science, Japan) (17). Dectin-2-deficient mice were viable and fertile without any visible signs of infections. Mice were kept in individually ventilated cages (Tecniplast) in a conventional facility. Our study examined male and female animals, and similar findings are reported for both sexes. All animal experiments were approved by the Animal Experimentation Review Board of Semmelweis University.

### Experimental EBA

Rabbits were immunized with a GST-CVII fusion protein (at Eurogentec) and polyclonal IgG (which contained antibodies against type VII collagen/referred to as anti-CVII antibodies/) was purified from the serum by an rProteinG-based (Thermo Scientific) chromatographic system. Experimental epidermolysis bullosa acquisita was induced by subcutaneous injections of anti-CVII antibodies as previously described (10, 11). In total 30 mg antibody was injected on day 0, 2 and 4 (10 mg/day), while control mice received PBS (Capricorn). Peripheral blood was collected from the tail on day 6 and serum anti-CVII levels were determined by a His-CVII-based ELISA as previously described (10, 18). During the experiment, clinical scores were given based on the body surface involvement and the nature of the lesions, while ear thickness was measured by a caliper (10, 11, 18). On day 14, after the photos were taken, the mice were euthanized. From the two ears of an animal, one was fixed in 4% paraformaldehyde, dehydrated, embedded in paraffin, while the other was used for flow cytometry (in order to quantify cell recruitement) and ELISA (for measuring cytokine concentrations; see below). Subsequently, 7-µm thick sections were stained with hematoxylin & eosin and were photographed by using a light microscope (Nikon) (11). The length of the visible dermal-epidermal separation was measured using the NIS-Elements Imaging Software.

For direct immunofluorescence, 4 mg of anti-CVII or normal rabbit IgG was injected subcutaneously into wild type and Dectin-2^–/–^ mice. After 24 hours, mice were euthanized and their ears were embedded in OCT (Sakura). 5-µm thick cryosections were stained with an Alexa Fluor 488-conjugated anti-rabbit antibody (Invitrogen) and were covered with a DAPI staining solution (Vector Laboratories). The antibody-deposition was evaluated under a fluorescent microscope (Nikon) as previously described (11, 18).

### Reverse passive Arthus reaction

Experimental Arthus reaction was induced in wild type and Dectin-2^–/–^ mice by the intravenous injection of 0.8 mg ovalbumin (Sigma-Aldrich) dissolved in 200 µl PBS (Capricorn) under isoflurane anesthesia (Baxter Hungary). The ears were injected intradermally with 40 µl anti-ovalbumin antibody-containing or control rabbit serum (Sigma-Aldrich and Capricorn, respectively) as previously described (19). After 2 hours, 200 µl of 0.5% Evans blue solution was administered retroorbitally. 0.5 and 24 hours later, ear thickness was measured by a caliper. Then the animals were euthanized, the ears were removed and were digested using Liberase (Sigma-Aldrich), stained with fluorochrome-conjugated anti-Ly6G (clone 1A8, BD Biosciences) and biotinylated anti-CD11b antibodies (clone M1/70, BD Biosciences) plus PerCP-Streptavidin (BD Biosciences). The numbers of accumulated double-positive neutrophils and CD11b^+^Ly6G^–^ macrophages were determined by flow cytometry (11). In the case of the modified Arthus reaction (“pseudo-Arthus”), ovalbumin was not used and anti-CVII antibodies were administered intradermally. Otherwise, the remaining steps were identical to those described in the case of the classical reverse passive Arthus reaction.

### *In vivo* migration assay

For the competitive migration assay, mixed bone marrow chimeras were generated. Wild type recipients were lethally irradiated by 11 Gy from a ^137^Cs source using a Gamma-Service Medical D1 irradiator, then were transplanted intravenously with a mixture of bone marrow cells from wild type donor mice carrying a CD45.1 allele and Dectin-2^–/–^ donor animals carrying a CD45.2 allele at different ratios as previously described (10). Experimental EBA was induced in these chimeras and on day 8 (after peripheral blood sampling), the mice were euthanized and their ear samples were digested with Liberase (Sigma-Aldrich). Both blood and ear samples were labeled with anti-CD45.2 (final concentration: 2 μg/ml, clone 104, BD Biosciences) and anti-Ly6G antibodies (final concentration: 0.8 μg/ml, clone 1A8, BD Biosciences). Double-positive cells were identified as Dectin-2^–/–^ neutrophils and the proportion of Dectin-2^–/–^ neutrophils in the peripheral blood and the inflamed tissues were compared. Based on these data, relative migration was calculated as previously described (20).

### Measuring the bone marrow and the circulating immune cell numbers

Bone marrow was washed out from the femurs and tibias using Hanks balanced salt solution (HBSS, Capricorn) containing 20 mM HEPES (Sigma-Aldrich). Red blood cells were removed by a hypotonic lysis and neutrophils were isolated by Percoll (GE Healthcare) gradient centrifugation as previously described (12, 20). Peripheral blood was taken from wild type or Dectin-2^–/–^ mice into phosphate buffer-based (PBS, Capricorn) solution containing 5% of fetal bovine serum (FBS, Capricorn) and 2% heparin-sodium (Teva) at 1:50.

Samples were stained with anti-Ly6G (final concentration: 0.8 μg/ml, clone 1A8) and biotinylated anti-CD11b antibodies (final concentration: 2 μg/ml, clone M1/70) plus PerCP-Streptavidin (all from BD Biosciences). As mentioned above, CD11b-positive and Ly6G-negative cells were identified as monocytes, while double-positive cells were identified as neutrophils by flow cytometry (BD FACSCalibur or Beckman Coulter CytoFLEX) as previously described (11, 21).

### *In vitro* activation of neutrophils

Mouse neutrophils were isolated from the bone marrow of the femurs and tibias of wild type mice by hypotonic lysis followed by Percoll (GE Healthcare) gradient centrifugation as previously described (see above) (12, 20). The cell surface expressions of various markers was detected on isolated or circulating neutrophils by anti-Ly6G-PE (final concentration: 0.8 μg/ml, clone 1A8, BD Biosciences), anti-FcγRIII (final concentration: 0.1 μg/ml, clone 275003, R&D Systems), anti-FcγRIV (final concentration: 0.8 μg/ml, clone 9E9, BD Biosciences), anti-CXCR2 (clone 242216, R&D Systems), anti-CD11a (final concentration: 2 μg/ml, clone M17/4, BD Biosciences), anti-CD11b (final concentration: 2 μg/ml, clone M1/70, BD Biosciences) or anti-CD18 antibodies (final concentration: 2 μg/ml, clone C71/16, BD Biosciences). Where required, primary antibodies were visualized by PerCP-Streptavidin (BD Biosciences). Flow cytometry was performed on a BD FACSCalibur or on a Beckman Coulter CytoFLEX.

Immobilized immune complex (IC) surfaces were obtained by coating the Nunc MaxiSorp F96 plates (Thermo Fisher) with human serum albumin (HSA, Sigma) at a 1:50 dilution, followed by a blocking step with fetal bovine serum (FBS, Capricorn) and a treatment with a polyclonal anti-human serum albumin antibody (anti-HSA, Sigma) at a 1:400 dilution as previously described (22, 23). For further validation, CVII and anti-CVII antibody-containing immune complexes were also generated. Here, Nunc MaxiSorp F96 plates (Thermo Fisher) were coated with His-CVII at a concentration of 20 µg/ml, were blocked with a 10% FBS solution (Capricorn), and were then coated with anti-CVII antibodies at a concentration of 300 µg/ml.

Bone marrow-derived wild type and Dectin-2^–/–^ neutrophils were plated on the immune complex-coated plates in the presence of 0.5 mM CaCl_2_ (Sigma-Aldrich) and 1 mM MgCl_2_ (Sigma-Aldrich) in Hanks balanced salt solution (HBSS, Capricorn) supplemented with 20 mM HEPES solution (Sigma-Aldrich) or in Dulbecco’s Modified Eagle Medium (DMEM, Capricorn) in the case of proinflammatory mediator concentration measurements. Neutrophil assays were performed at 37 °C. Superoxide release was measured by a cytochrome c (Sigma-Aldrich) reduction assay, cell spreading was followed by a phase contrast microscope (Leica) at a 20x magnification, while neutrophils were stimulated for 1 (in the case of LTB_4_) or 6 hours (in the case of cytokines) (12, 20, 21). In order to examine functionality, Nunc MaxiSorp F96 plates (Thermo Fisher) were coated with 10% FBS solution (Capricorn) and cells were treated with phorbol 12-myristate 13-acetate (PMA, Sigma-Aldrich) at a concentration of 10 µM (21).

For testing cell migration, wild type and Dectin-2^–/–^ neutrophils were placed in 3 µm pore-sized, fibrinogen-coated (Sigma-Aldrich) inserts of a Transwell plate (Corning) (12). The plate wells contained 3 µM fMLP (Sigma-Aldrich) or 100 ng/ml CXCL2 (PeproTech). The amount of transmigrated cells was measured by an acid phosphatase assay (12).

### Culturing and activating bone marrow-derived macrophages

The bone marrow was washed out with PBS (Capricorn) under sterile conditions, passed through a cell filter with a pore size of 70 µm, and then resuspended in a MEM-based solution (Biosera) containing 10% FBS (Capricorn) and 1% of Penicillin-Streptomycin (Capricorn) in the presence of 10 ng/ml recombinant murine macrophage colony-stimulating factor (M-CSF, PeproTech). Cells were placed in polystyrene petri dishes (Greiner) and two days later, the supernatant was centrifuged and the cells were resuspended in a fresh MEM-based (FBS- and antibiotic-containing) solution (Biosera), which was supplemented with the supernatant of M-CSF-producing CMG14–12 cells (20). A confluent macrophage culture was reached in a sterile incubator (Nuaire) at 37 °C as previously described (20).

Mature macrophages were placed in wells (in FluoroNunc MaxiSorp plates, Thermo Fisher) that were coated with HSA and anti-HSA antibody-containing immune complexes (see above) and reactive oxygen species production was detected by a luminometric method with a Varioskan LUX Multimode Microplate Reader (Thermo Fisher). For detecting cytokine production, cells were resuspended in DMEM (Capricorn) for 24 h at 37 °C (20).

### Detection of inflammatory mediators

Affected ears were digested by using Liberase (Sigma-Aldrich). The levels of inflammatory mediators in the supernatants of *in vivo* digested ear samples or of *in vitro* activated neutrophils and macrophages were measured by commercial ELISA kits (R&D Systems) according to the manufacturer’s instructions (10, 11).

### Statistical analysis

All experiments were performed at the indicated number of times. Statistical analysis was performed by the STATISTICA program using two-way ANOVA tests (with applying Tukey *post hoc* analysis). For kinetic datasets, the repeated measures ANOVA test was used with Tukey *post hoc* analysis. Kinetic curves and graphs show mean and standard error of the mean (SEM) from all independent *in vitro* experiments or from all individual mice of the different *in vivo* experiments. (Individual animals were considered as the experimental unit for both the *in vivo* and the *in vitro* assays.) P-values below 0.05 were considered statistically significant. The exact p-values are shown in the text.

## Results

### Dectin-2-deficiency did not influence the circulating or the bone marrow innate immune cell numbers and did not alter the maturation or the expression levels of Fcγ receptors or β_2_ integrins of neutrophils

First, we checked if the absence of Dectin-2 affected the circulating or the bone marrow numbers of neutrophils or monocytes/macrophages. We found that the bone marrow neutrophil or the bone marrow-derived macrophage numbers did not significantly differ in the case of wild type and Dectin-2-deficient mice, while the peripheral blood counts were also similar in the two genotypes (**Figure 1a**; p = 0.53, p = 0.49, p = 0.39 and p = 0.81, respectively). We also observed a normal maturation marker expression of neutrophils lacking Dectin-2 (**Figure 1b**). Fcγ receptors and β_2_ integrins are crucial mediators of experimental EBA (3, 6). Importantly, the expression levels of the activating FcγRIII and FcγRIV or the subunits of β_2_ integrins were comparable between the two genotypes, similar to the main chemokine receptor CXCR2 (**Figure 1b**).

**Figure 1:**
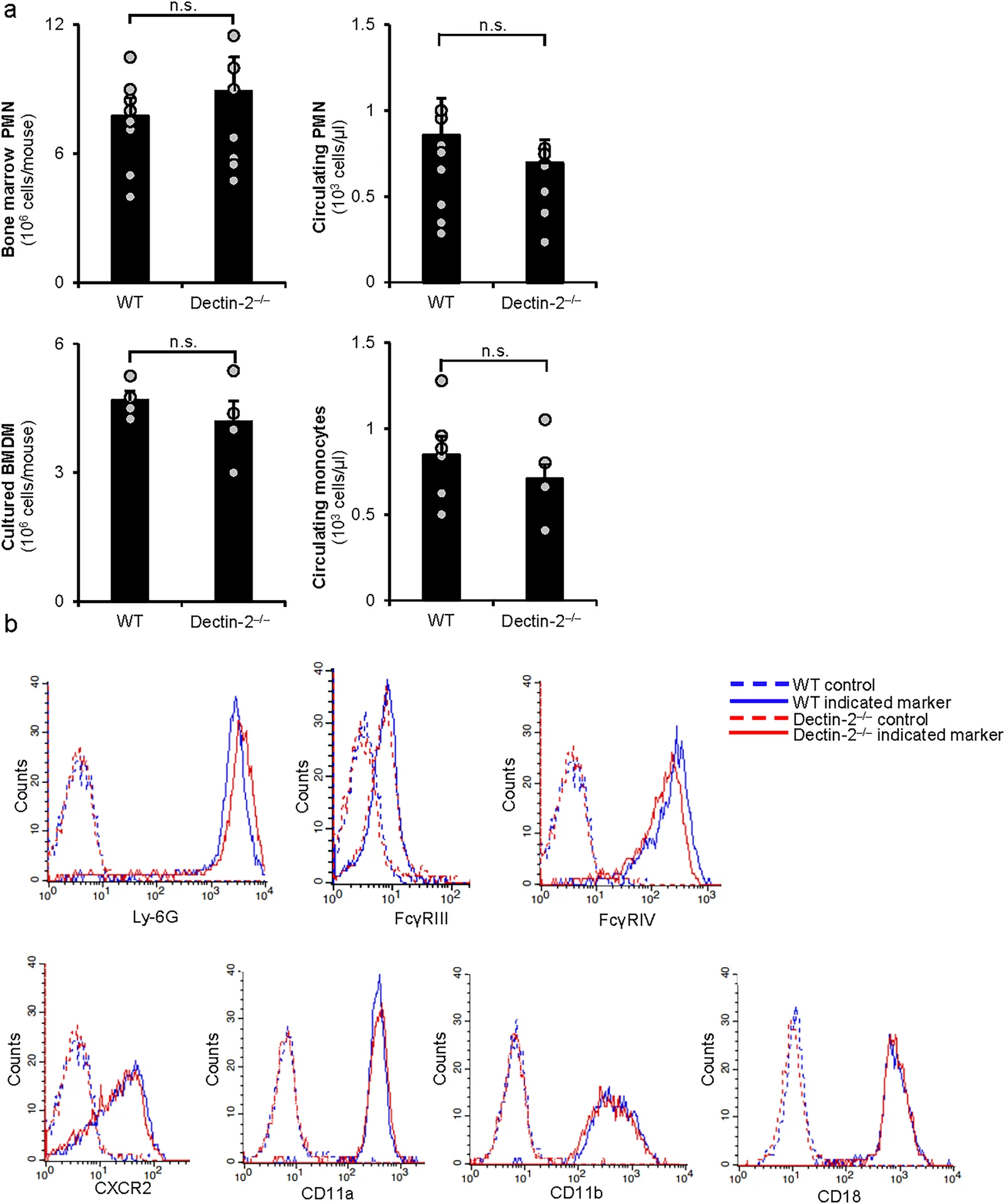
Characterization of Dectin-2^–/–^ mice. **(a)** Average bone marrow-isolated neutrophil (PMN) numbers, cultured bone marrow-derived macrophage (BMDM) numbers and circulating neutrophil and monocyte counts of WT and Dectin-2^–/–^ mice. **(b)** Cell surface marker expression of bone marrow-isolated neutrophils of WT and Dectin-2^–/–^ mice. Graphs in panel **(a)** show mean and SEM from 4 to 12 independent experiments. Panel **(b)** are representative images of 3 to 4 independent experiments. See the text for actual P-values. BMDM, bone marrow-derived macrophage; n.s., statistically non-significant. PMN, polymorphonuclear cell; SEM, standard error of the mean; WT, wild type.

### Dectin-2-deficient mice were protected from the development and progression of experimental epidermolysis bullosa acquisita

When wild type animals received anti-CVII antibodies intradermally to their ears, the ears showed blue discoloration after the intravenous administration of Evans blue solution and a massive thickening both at 2.5 and 24 hours (**Figures 2a, b**). Here, CVII and anti-CVII antibody-containing immune complexes triggered a fast and robust inflammatory reaction leading to increased vascular permeability and the extravasation of the albumin-bound Evans blue dye, causing ear thickness changes. In sharp contrast to wild type mice, Dectin-2-deficient animals had reduced discoloration of the antibody-injected ear and the thickness changes were significantly reduced (**Figures 2a, b**; p = 0.005 and p = 2.5 x 10^-4^ /WT anti-CVII vs. Dectin-2^–/–^ anti-CVII/, respectively). Furthermore, neutrophil recruitment and CXCL2 levels in the ears were massively reduced in the absence of Dectin-2 (**Supplementary Figures 1a, b**; p = 0.018 and p = 0.009 /WT anti-CVII vs. Dectin-2^–/–^ anti-CVII/, respectively).

**Figure 2:**
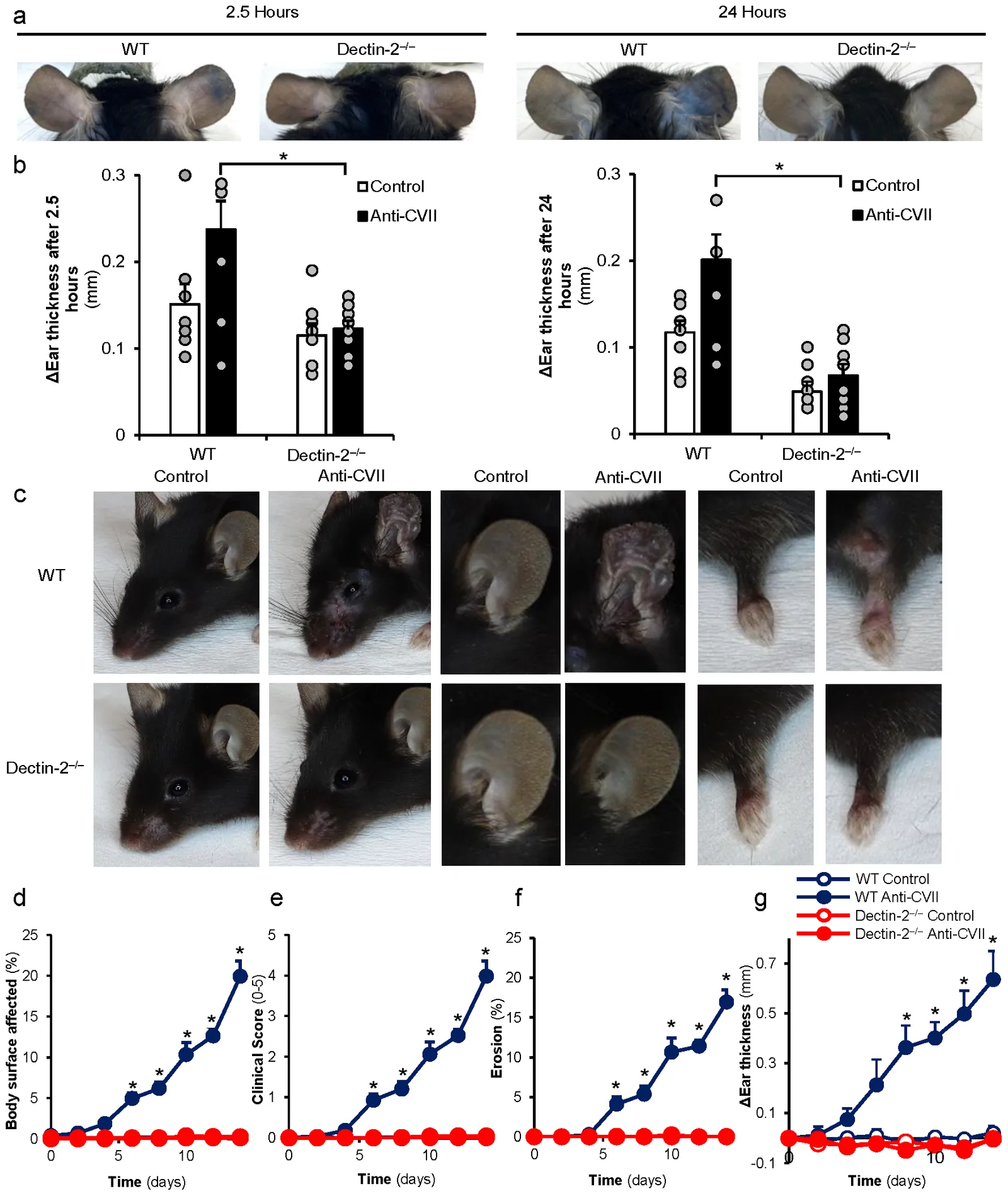
Experimental EBA in Dectin-2^–/–^ mice. **(a, b)** A modified version of reverse passive Arthus reaction (“pseudo-Arthus”) was monitored in WT and Dectin-2^–/–^ mice by taking representative photos 2.5 hours and 24 hours after induction **(a)** and measuring ear thickness changes at two timepoints **(b)**. Experimental EBA was induced in WT and Dectin-2^–/–^ mice and followed by photodocumentation at day 14 **(c)**, affected body surface extent **(d)**, clinical scoring **(e),** the extent of erosions **(f)** and ear thickness measurements **(g)**. Graphs in panel **(b)** show mean and SEM from 8 mice per genotype from 4 independent experiments. Graphs in panel **(d–g)** show mean and SEM from 4–7 control and 5–10 anti-CVII-treated mice per genotype from 3–4 independent experiments. Panels **(a)** shows representative images from 8 mice per genotype from 4 independent experiments. Panels **(c)** contains representative images from 7 control and 10 anti-CVII-treated mice per genotype from 4 independent experiments. See the text for actual P-values. Anti-CVII; anti-type VII collagen antibody; EBA, epidermolysis bullosa acquisita; SEM, standard error of the mean; WT, wild type; *p < 0.05.

In line with these observations, wild type mice treated with repeated subcutaneous injections of anti-CVII antibodies developed visible skin inflammation on the head and on the hind limbs, while the absence of Dectin-2 led to a near total protection from these observable macroscopic alterations (**Figure 2c**). The percentage of the affected body surface area gradually increased in wild type animals, whereas nearly no signs of the disease could be observed in Dectin-2-deficient mice (**Figure 2d**; *p < 0.05 /WT anti-CVII vs. Dectin-2^–/–^ anti-CVII/). The clinical score, the number of the dermal-epidermal separation-derived erosions and the ear thickness also gradually increased during the assay period in wild type mice without any significant signs of the disease in Dectin-2-deficient animals (**Figures 2e–g**; * p < 0.05 /WT anti-CVII vs. Dectin-2^–/–^ anti-CVII/).

Taken together, the absence of Dectin-2 reduced the inflammatory signs of anti-CVII antibody-induced experimental dermatitis without affecting the peripheral and the bone marrow innate immune cell numbers or the maturation of neutrophils and the expression levels of critical (neutrophil) receptors required for experimental EBA.

### Dectin-2-deficiency also had a protective effect in the microscopic appearance of inflammation, but did not affect the circulating anti-CVII antibody levels and the deposition of these antibodies to the dermal-epidermal junction

Next, we made histological sections of the ears of wild type and Dectin-2-deficient mice. As shown in **Figure 3a**, while anti-CVII antibody-treated wild type animals had a massive immune cell infiltration in their ears causing significant thickening, mice lacking Dectin-2 had (almost) intact tissues without any visible signs of inflammation. Furthermore, a high percentage of dermal-epidermal separation (which is a classical EBA manifestation leading to blisters and erosions) was observable in skin histological sections of wild type, but not in Dectin-2-deficient mice (**Figure 3a**; p = 2.6 x 10^-4^ /WT anti-CVII vs. Dectin-2^–/–^ anti-CVII/). Meanwhile, the circulating anti-CVII antibody levels did not differ between the two genotypes and the deposition of the antibodies to the dermal-epidermal junction was also unaffected by the presence of Dectin-2 (**Figures 3b, c**; p = 0.74 /WT anti-CVII vs. Dectin-2^–/–^ anti-CVII/for the ELISA). These data show that Dectin-2 is not required for the elimination of the antibodies from the peripheral blood or the presence of the antibodies in the skin, suggesting that the role of Dectin-2 lines in the events that follow the deposition of the pathogenic antibodies to the dermal-epidermal junction.

**Figure 3:**
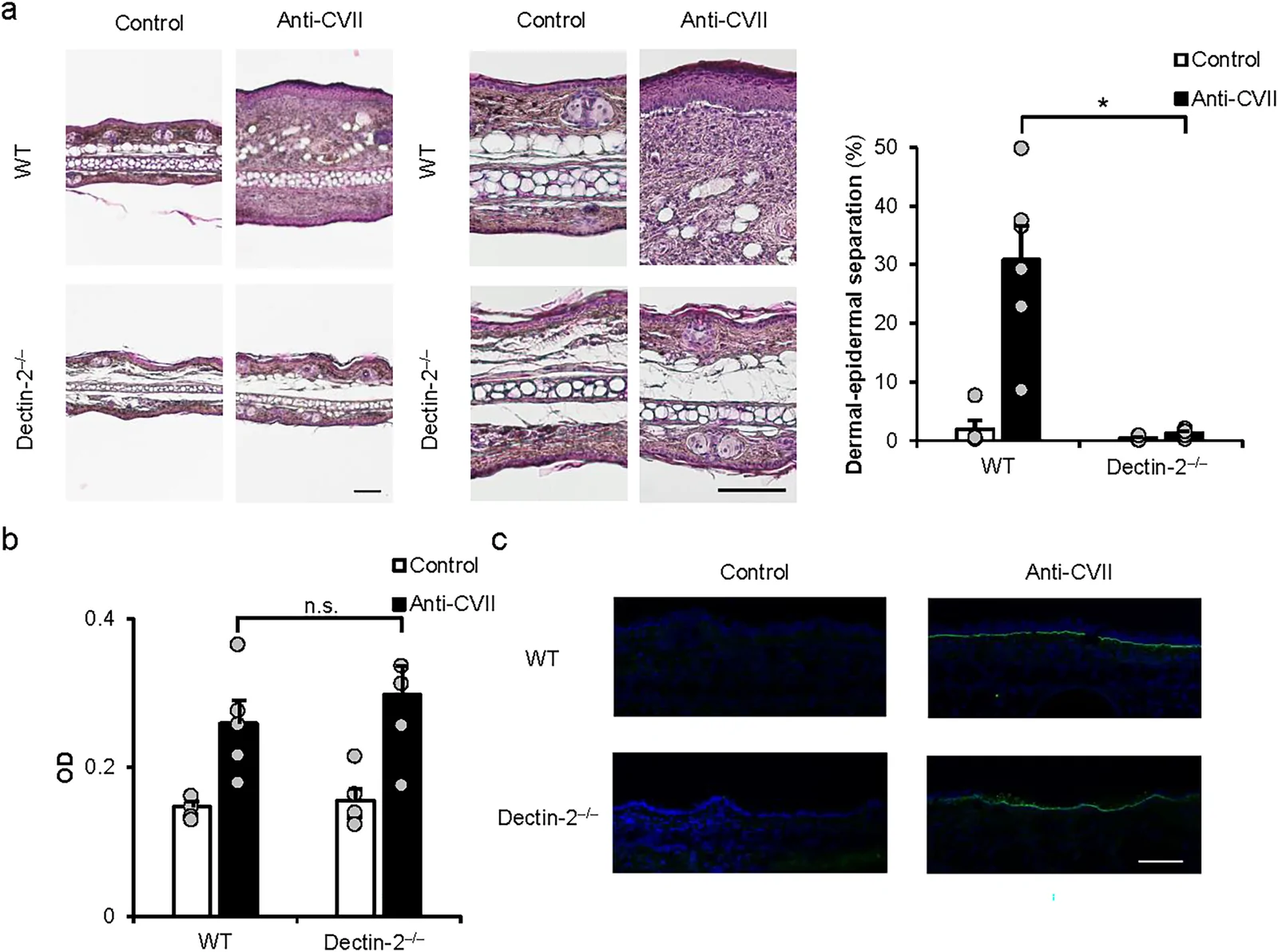
Microscopic phenotype of experimental EBA in Dectin-2^–/–^ mice. **(a)** Histological analysis of the ears of WT and Dectin-2^–/–^ mice at 4x and 10x magnification, and the quantification of the dermal-epidermal separation. **(b)** Peripheral anti-CVII antibody levels of WT and Dectin-2^–/–^ mice. **(c)** Anti-CVII antibody deposition in the ears of WT and Dectin-2^–/–^ mice. Graph in panel **(a)** shows mean and SEM from 4–5 control and 5–6 anti-CVII-treated mice per genotype from 3 independent experiments. Graph in panel **(b)** shows mean and SEM from 5 independent experiments. Panels **(a, c)** contain representative images from 3 independent experiments. See the text for actual P-values. Anti-CVII; anti-type VII collagen antibody; n.s., statistically non-significant; OD, optical density; SEM, standard error of the mean; WT, wild type; *p < 0.05. Scale bar: 100 µm.

### Dectin-2 is required for the generation of the inflammatory environment

Several immune cells – like neutrophils and monocytes/macrophages – participate in the pathogenesis of EBA (24). When we measured the neutrophil and macrophage numbers at the site of inflammation, we observed a massive increase in the cellular counts in anti-CVII antibody-treated wild type mice compared to the control animals, which was totally absent in connection with Dectin-2-deficient mice (**Figures 4a, b**; p = 1.9 x 10^-2^ and p = 5.5 x 10^-3^ /WT anti-CVII vs. Dectin-2^–/–^ anti-CVII/, respectively). Furthermore, we detected dramatically reduced LTB_4_ levels in the lack of Dectin-2, while the concentrations of cytokines/chemokines (e.g. IL-1β, CXCL2 and CCL3) were also massively decreased (**Figure 4c**; p = 2.2 x 10^-2^, p = 2.3 x 10^-2^, p = 3.5 x 10^-2^ and p = 4.3 x 10^-2^ /WT anti-CVII vs. Dectin-2^–/–^ anti-CVII/, respectively). These results show that Dectin-2 is indispensable for the generation of the inflammatory environment during the development of experimental EBA.

**Figure 4:**
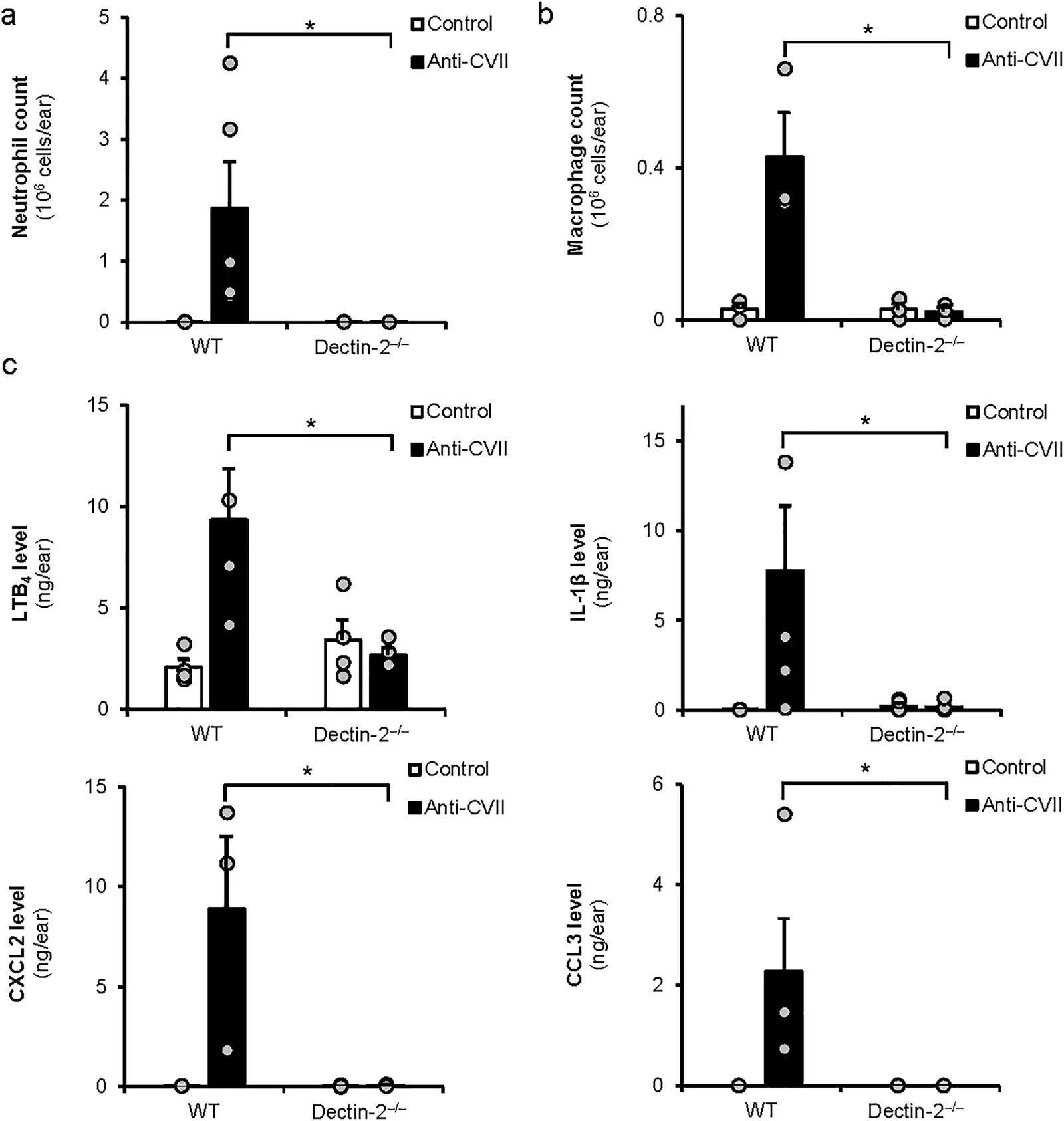
*In vivo* proinflammatory microenvironment of experimental EBA in Dectin-2^–/–^ mice. *In vivo* neutrophil **(a)** and macrophage **(b)** accumulation at the site of inflammation of WT and Dectin-2^–/–^ mice. **(c)** *In vivo* proinflammatory mediator levels of WT and Dectin-2^–/–^ mice. Graphs in panels **(a–c)** show mean and SEM from 3–5 independent experiments. See the text for actual P-values. Anti-CVII, anti-type VII collagen antibody; CCL3, C-C motif chemokine ligand 3; CXCL2, C-X-C motif chemokine ligand 2; IL-1β, interleukin-1β; LTB_4_, leukotriene B_4_; SEM, standard error of the mean; WT, wild type; *p < 0.05.

### Dectin-2 is not required for the *in vitro* and *in vivo* migration of neutrophils

In order to test the *in vitro* migration of wild type and Dectin-2-deficient neutrophils, we used the Transwell assay, where neutrophils in the upper compartment (in the insert) had to undergo active morphological changes to integrin-dependently reach the lower compartment (the well), which contained the chemoattractants. Both when we used the chemokine CXCL2 or the bacterial tripeptide fMLP, wild type cells and neutrophils lacking Dectin-2 could migrate though the 3 µm pore size and could reach the bottom of the lower compartment at similar quantities (**Figures 5a, b**; p = 0.87 /WT CXCL2 vs. Dectin-2^–/–^ CXCL2/and p = 0.81/WT fMLP vs. Dectin-2^–/–^ fMLP/, respectively).

**Figure 5:**
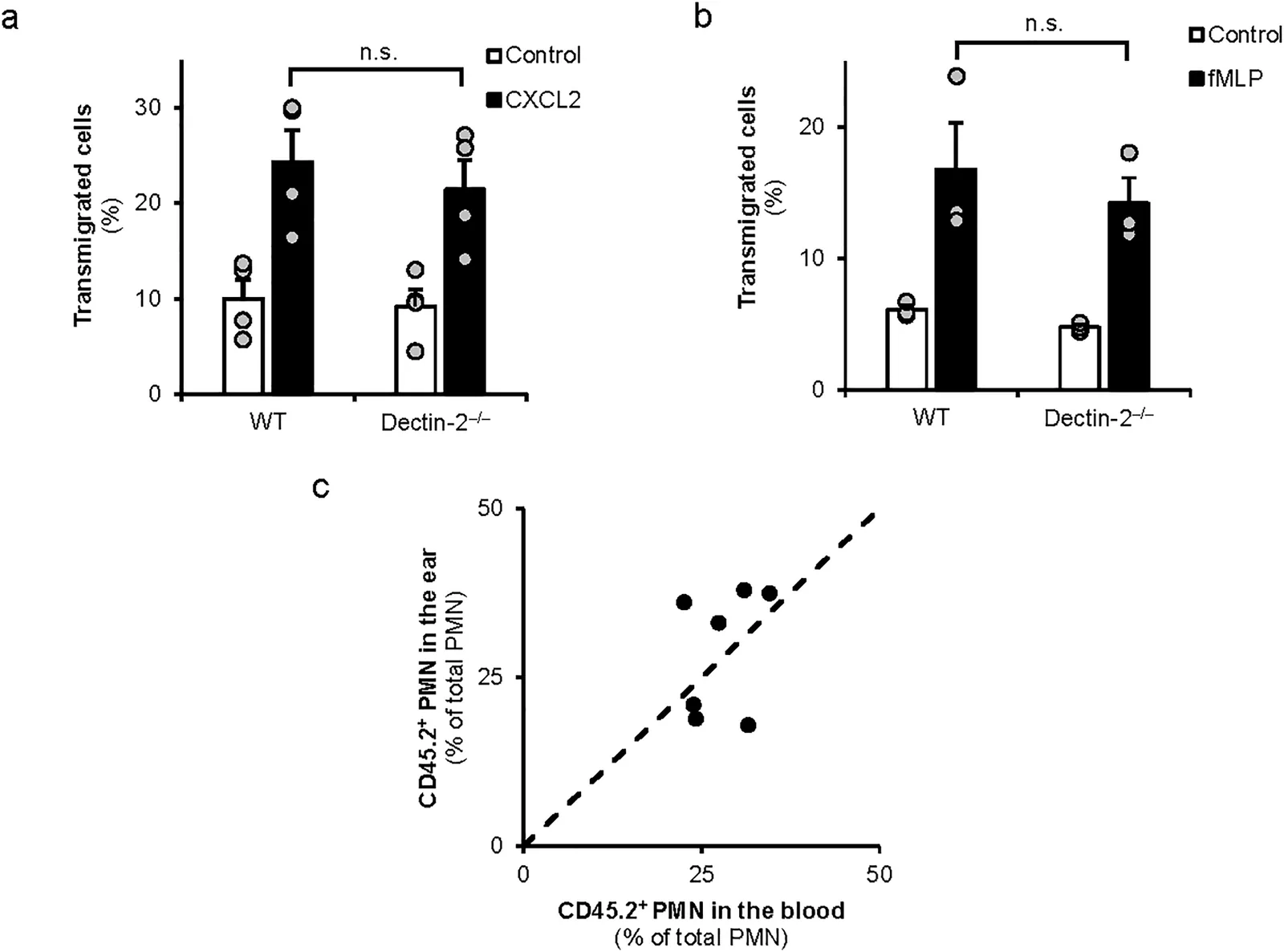
Migration capacity of Dectin-2^–/–^ neutrophils. *In vitro* migration of WT and Dectin-2^–/–^ neutrophils triggered by CXCL2 **(a)** or fMLP **(b)**. **(c)** *In vivo* migration of Dectin-2^–/–^ neutrophils in mixed WT/Dectin-2^–/–^ bone marrow chimeric mice in experimental EBA. Graphs in panel **(a, b)** show mean and SEM from 3–4 independent experiments. In panel **(c)**, each dot represents an individual mouse from 7 mice from 3 independent experiments. See the text for actual P-values. CXCL2, C-X-C motif chemokine ligand 2; fMLP, N-formylmethionine leucyl-phenylalanine; n.s., statistically non significant; PMN, polymorphonuclear cell; SEM, standard error of the mean; WT, wild type.

We also generated mixed bone marrow chimeras with different ratios of CD45.2-negative wild type and CD45.2-positive Dectin-2-deficient myeloid cells and triggered experimental EBA in these animals. After the development of inflammation, we measured the percentage of CD45.2-negative and -positive cells in the ear tissue and in the peripheral blood. When we compared these ratios, we found that Dectin-2-deficient neutrophils could as efficiently migrate to the ear as wild type cells, meaning that Dectin-2 was also not important for *in vivo* migration (**Figure 5c**; p = 0.54).

These data suggested that the role of Dectin-2 needed to be further investigated in the postmigratory effector functions of neutrophils.

### Dectin-2 is essential for immune complex-mediated neutrophil, but not for macrophage functions

When we plated freshly isolated bone marrow-derived neutrophils on HSA–anti-HSA antibody containing immune complexes, we observed a massive reduction in the superoxide release of Dectin-2-deficient neutrophils compared to wild type cells (**Figure 6a**; *p < 0.05 /WT IC vs. Dectin-2^–/–^ IC/). However, when we used serum-coated wells and the protein kinase C (PKC) activator PMA, both genotypes produced similar amounts of toxic oxygen intermediers, indicating that the lack of Dectin-2 did not alter cellular functionality (**Figure 6b**; n.s. at all timepoints/WT PMA vs. Dectin-2^–/–^ PMA/). Further experiments showed that Dectin-2 was also required for immune complex-triggered neutrophil spreading, lipid mediator (LTB_4_) and chemokine (CXCL2) production (**Figures 6c, d**; p = 2.3 x 10^-3^, p = 2.7 x 10^-2^ and p= 2.9 x 10^-2^ /WT IC vs. Dectin-2^–/–^ IC/, respectively).

**Figure 6:**
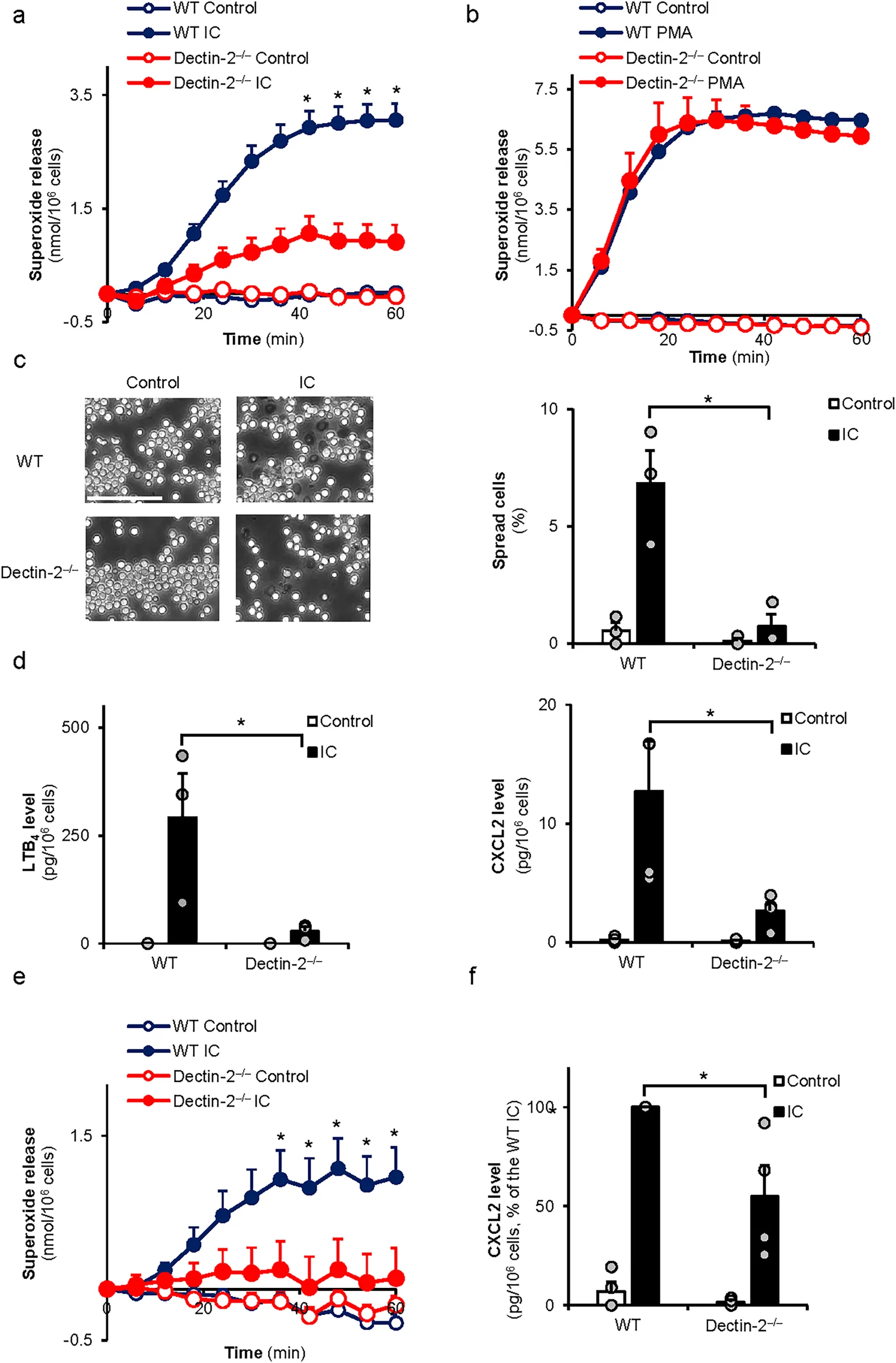
Effector functions of immune complex-mediated Dectin-2^–/–^ neutrophils. Superoxide-production of HSA-αHSA IC- **(a)** or PMA-stimulated **(b)** WT and Dectin-2^–/–^ neutrophils. **(c)** Representative images and quantification of cell spreading of WT and Dectin-2^–/–^ neutrophils after HSA-αHSA IC-stimulation. **(d)** Proinflammatory mediator-release of HSA-αHSA IC-stimulated WT and Dectin-2^–/–^ neutrophils. Superoxide-release **(e)** and cytokine-production **(f)** of WT and Dectin-2^–/–^ neutrophils stimulated by CVII–anti-CVII IC surfaces. Graphs in all panels show mean and SEM from 3–6 independent experiments. Panels **(c)** shows representative images from 3 independent experiments. See the text for actual P-values. CXCL2, C-X-C motif chemokine ligand 2; IC, immune complex; LTB_4_, leukotriene B_4_; PMA, phorbol 12-myristate 13-acetate; SEM, standard error of the mean; WT, wild type; *p < 0.05. Scale bar: 50 µm.

As we wanted to test if the above findings were related only to HSA–anti-HSA antibody containing immune complexes, we also used CVII–anti-CVII (“EBA”) immune complexes. Similar to the other immune complex-coated surface, wild type neutrophils produced high amounts of superoxide and CXCL2 when plated on EBA immune complexes, however, Dectin-2-deficient neutrophils again had reduced cell responses (**Figure 6e**; *p < 0.05 /WT IC vs. Dectin-2^–/–^ IC/; **Figure 6f**; p = 8.9 x 10^-3^ /WT IC vs. Dectin-2^–/–^ IC/, respectively).

Given the importance of macrophages in the pathogenesis of EBA, we cultured macrophages from the bone marrow and tested their maturation marker and activating Fcγ receptor expression in the presence and absence of Dectin-2 (**Supplementary Figure 1c**). As expected, we did not observe any major differences in the expression levels of the maturation marker F4/80, FcγRIII and FcγRIV of wild type and Dectin-2-deficient macrophages (**Supplementary Figure 1c**). Next, we plated these immune cells on immobilized immune complex surfaces. Interestingly, macrophages lacking Dectin-2 produced similar amounts of superoxide and CXCL2 than wild type cells (**Supplementary Figure 1d**; n.s. at all timepoints /WT IC vs. Dectin-2^–/–^ IC/; **Supplementary Figure 1e**; p = 0.79 /WT IC vs. Dectin-2^–/–^ IC/, respectively).

These data suggest that – in contrast to macrophages – Dectin-2 was important for Fcγ receptor-mediated neutrophil cell responses.

### The presence of Dectin-2 is critical for the immune complex- and neutrophil-mediated reverse passive Arthus-reaction

Next, we were interested if Dectin-2 was also important in the development of another immune complex-driven *in vivo* animal model, namely in the reverse passive Arthus-reaction. Upon the formation of ovalbumin–anti-ovalbumin immune complexes in one ear, in contrast to the other ear, the intravenously injected albumin-binding Evans blue dye colored the tissue of wild type animals after 2.5 and 24 hours, showing a rise in the permeability of the small vessels due to the first steps of inflammation (**Figures 7a, b**). In sharp contrast to wild type animals, Dectin-2-deficient mice showed a massively reduced macroscopic phenotype, where the immune complex-treated ears were just slightly or not affected (**Figures 7a, b**). When measuring the ear thickness, we observed a very similar phenomenon: a robust increase in wild type, but not in Dectin-2 knockout animals at both timepoints (**Figure 7c**; p = 1.6 x 10^-4^ and p = 1.6 x 10^-4^ /WT anti-OVA vs. Dectin-2^–/–^ anti-OVA/, respectively). In line with these findings, 24 hours after the induction of inflammation, neutrophil recruitment, but also tissue C5a levels were dramatically decreased in mice lacking Dectin-2 (**Figures 7d, e**; p = 9.2 x 10^-4^ and p = 3.8 x 10^-2^ /WT anti-OVA vs. Dectin-2^–/–^ anti-OVA/, respectively). These data show that Dectin-2 is crucial in the very first and early steps of immune complex-mediated inflammation. Here, we could show that Dectin-2 is also a critical component of another immune complex-driven model, validating our above mentioned results with experimental EBA.

**Figure 7:**
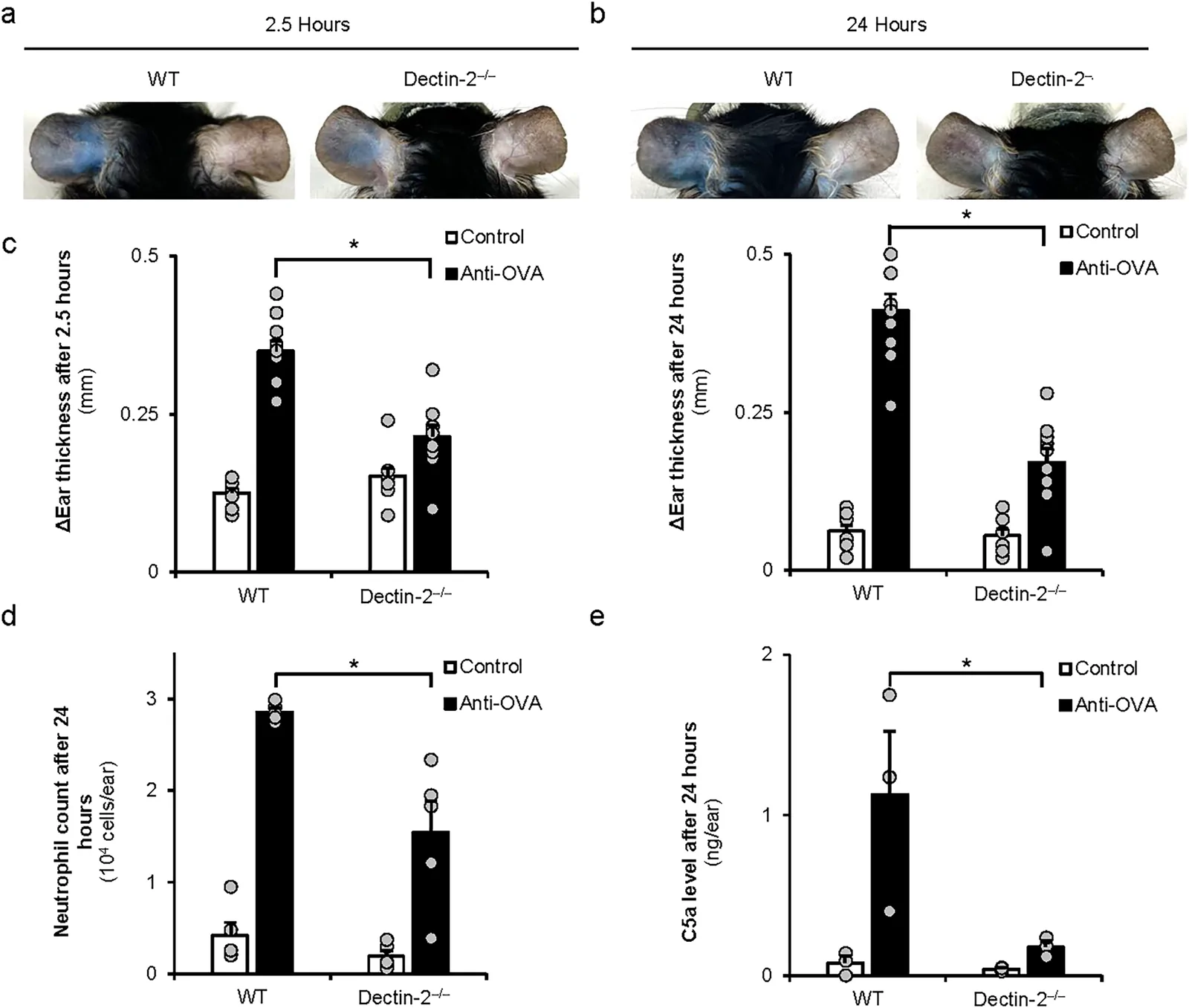
Reverse passive Arthus reaction in Dectin-2^–/–^ mice. Representative pictures of WT and Dectin-2^–/–^ mice 2.5 hours **(a)** and 24 hours **(b)** after the disease induction. **(c)** Changes of the ear thickness of WT and Dectin-2^–/–^ mice after 2.5 and 24 hours. **(d)** Neutrophil cell recruitment to the ears of WT and Dectin-2^–/–^ mice 24 hours after disease induction. **(e)** C5a levels in the ears of WT and Dectin-2^–/–^ mice after 24 hours. Graphs in panel **(c–e)** show mean and SEM from 6–10 mice per genotype from 3–5 independent experiments. Panels **(a, b)** show representative images from 10 mice per genotype from 5 independent experiments. See the text for actual P-values. Anti-OVA, anti-ovalbumin antibody; C5a, complement component 5a; SEM, standard error of the mean; WT, wild type; *p < 0.05.

Overall, our results demonstrated that Dectin-2 is a critical component of experimental immune complex-mediated *in vivo* inflammation, which is probably based on the role of this C-type lectin receptor in postmigratory neutrophil cell responses. These data also indicate that Dectin-2 could serve a therapeutic target in the treatment of these disorders in the future.

## Discussion

Here we report that the C-type lectin receptor Dectin-2 is essential for the development and progression of the neutrophil-dependent experimental epidermolysis bullosa acquisita, from a very early phase (**Figure 2**). We showed that the lack of Dectin-2 blocked the dermal-epidermal separation at lesional sites, while it did not affect circulating anti-type VII collagen antibody levels and the deposition of the antibodies to the dermal-epidermal junction (**Figure 3**). Meanwhile, neutrophil and monocyte/macrophage recruitment to the site of skin inflammation was dramatically reduced in Dectin-2-deficient mice with massively decreased (dominantly neutrophil-derived) local lipid, cytokine and chemokine levels (**Figure 4**). This phenomenon could not be explained neither by reduced circulating neutrophil or monocyte counts, their affected cell surface expression of Fcγ receptors, integrins or the chemokine receptor CXCR2, nor by an altered migratory capacity of neutrophils (**Figures 1**, **5**). These data are consistent with previous findings, which showed that the Dectin-2 encoding *Clec4n* gene was found to be upregulated in neutrophils in the lesional skin in experimental and human EBA (16).

In addition, another immune complex- and neutrophil-dependent *in vivo* model, namely the reverse passive Arthus reaction was also significantly affected by the absence of Dectin-2, which was also characterized by a defect in neutrophil accumulation and C5a generation/release (**Figure 7**) (20). In parallel with these observations, the anti-GPI antibody-dependent K/BxN serum transfer arthritis also showed a critical Dectin-2-dependency, which was measured by our collaborators (Valentova et al., manuscript submitted). This means that Dectin-2 was a crucial element for three different, mainly immune complex- and neutrophil-mediated experimental models, highlighting that the normal expression of Dectin-2 on neutrophils is potentially indispensable for these Fcγ receptor-dependent processes.

We also found that Dectin-2 was required for the immune complex-mediated *in vitro* neutrophil cell responses like superoxide release (which is crucial for dermal-epidermal separation), cell spreading and lipid mediator/chemokine production, while it was not important for integrin-dependent migration (**Figures 5**, **6**). This proposes that Dectin-2-deficient neutrophils could reach the site of anti-CVII antibody deposition at the dermal-epidermal junction, but fail to undergo Fcγ receptor-mediated cell activation, which is crucial for local tissue damage (leading to blisters and erosions) and for recruiting further neutrophils and other immune cells (which blocks the positive feedback amplification loop in the case of LTB_4_ and CXCL2 in connection with neutrophils) (10–12, 25). In the light of these findings, it is rather surprising that the neutrophil-specific deletion of Dectin-2 (with the promoter MRP8) did not result in an alteration in the EBA phenotype (16). This could be due to the incomplete deletion of Dectin-2 from neutrophils and/or the technical difference in the management and induction of experimental EBA. For instance, while the MRP8 promoter-driven Cre recombinase is already active in a proportion of granulocyte/macrophage progenitors, it does not cause a total elimination of the target protein from mature neutrophils (which we also experienced previously) (12, 26). Another important fact is that while we kept our mice and induced experimental EBA in a conventional animal facility, Christian F. Guerrero-Juarez and his colleagues housed their mice under specific pathogen-free conditions, which may account for the different EBA phenotypes of the Dectin-2 knockout transgenic mice due to the alterations in the microbiome (especially if we take into account that Dectin-2 is a pattern recognition receptor) (16). It should be also noted that while we used polyclonal IgG preparations in our protocol, the other workgroup utilized affinity-purified antibodies for the induction process (16). However, the massive phenotypes in our two independent, mainly neutrophil-dependent *in vivo* models in mice lacking Dectin-2 and a similar phenotype in the heavily neutrophil-mediated K/BxN serum transfer arthritis (observed by our collaborators) raise the potential importance of Dectin-2 in the neutrophil compartment in immune complex-triggered inflammation.

As we used two different types of immune complexes (i.e. HSA–anti-HSA and CVII–anti-CVII immune complexes) with a rabbit origin in the above shown experiments and performed further *in vitro* neutrophil assays on a third one, which contained the mouse-derived anti-GPI antibody (Valentova et al., manuscript submitted), we propose that the observed defect of neutrophil cell responses in the lack of Dectin-2 is not exclusively dependent on one particular type of immune complex, but is rather a general phenomenon of Fcγ receptor-mediated neutrophil functions in the absence of Dectin-2.

While the direct binding capability of Dectin-2 to immune complexes has not been proved yet, it is a probable scenario that this C-type lectin receptor – which may bind to the carbohydrate moieties (especially to the mannose-rich regions) of IgG antibodies – could also attach to the antibodies in formed immune complexes and augments the cell responses triggered through Fcγ receptors, which could explain the *in vivo* and the *in vitro* defects seen in the absence of the molecule (27, 28). A further important fact is that mouse activating Fcγ receptors and Dectin-2 use a similar signaling machinery with the involvement of the Fc receptor γ-chain, the Syk tyrosine kinase and CARD9, which have been shown to be important in the development of experimental autoimmune arthritis and dermatitis (9, 10, 12, 29–31). Moreover, we have previously shown that Syk and CARD9 are important molecules especially in neutrophils during experimental EBA, further supporting a crucial role for Dectin-2 especially in the neutrophil compartment (11, 12).

Besides neutrophils, Dectin-2 is also expressed by other immune cells like dendritic cells or monocytes/macrophages (32). While dendritic cells are not important in the pathogenesis of experimental EBA or reverse passive Arthus reaction, monocytes/macrophages have a significant role in immune complex-mediated inflammation (33, 34). However, our models seem to be rather dependent on neutrophils and Dectin-2-deficient macrophages had normal immune complex-mediated cell responses in our hands and also in the laboratory of our international collaborators (**Supplementary Figure 1** and Valentova et al., manuscript submitted) (7, 35–37). As immune complex-stimulated neutrophils also showed a significantly reduced CCL3 production in the absence of Dectin-2 in our *in vitro* experiments, it is a possible scenario that the defect of Dectin-2-deficient neutrophils indirectly stands for the reduced macrophage accumulation seen in experimental EBA (**Figure 4** and data not shown).

Overall, we found that Dectin-2 is an essential component in immune complex-mediated inflammation – probably by acting in neutrophils and affecting their postmigratory immune complex-mediated cellular functions –, which raises the possibility that this C-type lectin receptor may serve as a potential therapeutic target in the control of such disorders (like autoimmune blistering skin diseases) in the future.

## Data Availability

The raw data supporting the conclusions of this article will be made available by the authors, without undue reservation.

## References

[1] Akbarialiabad H Schmidt E Patsatsi A Lim YL Mosam A Tasanen K . Bullous pemphigoid. Nat Rev Dis Primers. (2025) 11:12. doi: 10.1038/s41572-025-00595-5 39979318

[2] Kridin K Kneiber D Kowalski EH Valdebran M Amber KT . Epidermolysis bullosa acquisita: a comprehensive review. Autoimmun Rev. (2019) 18:786–95. doi: 10.1016/j.autrev.2019.06.007 31181325

[3] Sitaru C Mihai S Otto C Chiriac MT Hausser I Dotterweich B . Induction of dermal-epidermal separation in mice by passive transfer of antibodies specific to type VII collagen. J Clin Invest. (2005) 115:870–8. doi: 10.1172/jci200521386 15841176 PMC1070403

[4] Sitaru C . Experimental models of epidermolysis bullosa acquisita. Exp Dermatol. (2007) 16:520–31. doi: 10.1111/j.1600-0625.2007.00564.x 17518993

[5] Mihai S Chiriac MT Takahashi K Thurman JM Holers VM Zillikens D . The alternative pathway of complement activation is critical for blister induction in experimental epidermolysis bullosa acquisita. J Immunol. (2007) 178:6514–21. doi: 10.4049/jimmunol.178.10.6514 17475881

[6] Chiriac MT Roesler J Sindrilaru A Scharffetter-Kochanek K Zillikens D Sitaru C . NADPH oxidase is required for neutrophil-dependent autoantibody-induced tissue damage. J Pathol. (2007) 212:56–65. doi: 10.1002/path.2157 17380558

[7] Csepregi JZ Orosz A Zajta E Kása O Németh T Simon E . Myeloid-specific deletion of Mcl-1 yields severely neutropenic mice which survive and breed in homozygous form. J Immunol. (2018) 201:3793–803. doi: 10.4049/jimmunol.1701803 30464050 PMC6287103

[8] Sato K Yang XL Yudate T Chung JS Wu J Luby-Phelps K . Dectin-2 is a pattern recognition receptor for fungi that couples with the Fc receptor γ chain to induce innate immune responses. J Biol Chem. (2006) 281:38854–66. doi: 10.1074/jbc.m606542200 17050534

[9] Robinson MJ Osorio F Rosas M Freitas RP Schweighoffer E Gross O . Dectin-2 is a Syk-coupled pattern recognition receptor crucial for Th17 responses to fungal infection. J Exp Med. (2009) 206:2037–51. doi: 10.1084/jem.20082818 PMC2737172 19703985

[10] Németh T Virtic O Sitaru C Mócsai A . The Syk tyrosine kinase is required for skin inflammation in an *in vivo* mouse model of epidermolysis bullosa acquisita. J Invest Dermatol. (2017) 137:2131–9. doi: 10.1016/j.jid.2017.05.017 PMC5624865 28576735

[11] Németh T Balogh L Káposztás E Szilveszter KP Mócsai A . Neutrophil-specific Syk expression is crucial for skin disease in experimental epidermolysis bullosa acquisita. J Invest Dermatol. (2023) 143:1147–56. doi: 10.1016/j.jid.2022.12.016 36641133

[12] Németh T Futosi K Sitaru C Ruland J Mócsai A . Neutrophil-specific deletion of the CARD9 gene expression regulator suppresses autoantibody-induced inflammation *in vivo*. Nat Commun. (2016) 7:11004. doi: 10.1038/ncomms11004 PMC4821996 27032818

[13] Bournazos S Ravetch JV . Diversification of IgG effector functions. Int Immunol. (2017) 29:303–10. doi: 10.1093/intimm/dxx025 28472280 PMC5890892

[14] Chiffoleau E . C-type lectin-like receptors as emerging orchestrators of sterile inflammation represent potential therapeutic targets. Front Immunol. (2018) 9:227. doi: 10.3389/fimmu.2018.00227 29497419 PMC5818397

[15] Caliz R Canet LM Lupianez CB Canhao H Escudero A Filipescu I . Gender-specific effects of genetic variants within Th1 and Th17 cell-mediated immune response genes on the risk of developing rheumatoid arthritis. PloS One. (2013) 8:e72732. doi: 10.1371/journal.pone.0072732 PMC3758336 24023637

[16] Guerrero-Juarez CF Schilf P Li J Zappia MP Bao L Patel PM . C-type lectin receptor expression is a hallmark of neutrophils infiltrating the skin in epidermolysis bullosa acquisita. Front Immunol. (2023) 14:1266359. doi: 10.3389/fimmu.2023.1266359 37799716 PMC10548123

[17] Saijo S Ikeda S Yamabe K Kakuta S Ishigame H Akitsu A . Dectin-2 recognition of α-mannans and induction of Th17 cell differentiation is essential for host defense against Candida albicans. Immunity. (2010) 32:681–91. doi: 10.1016/j.immuni.2010.05.001 20493731

[18] Szilveszter KP Vikár S Horváth ÁI Helyes Z Sárdy M Mócsai A . Phospholipase Cγ2 is essential for experimental models of epidermolysis bullosa acquisita. J Invest Dermatol. (2022) 142:1114–25. doi: 10.1016/j.jid.2021.09.019 34656615

[19] Orosz A Walzog B Mócsai A . *In vivo* functions of mouse neutrophils derived from HoxB8-transduced conditionally immortalized myeloid progenitors. J Immunol. (2021) 206:432–45. doi: 10.4049/jimmunol.2000807 33310871

[20] Kovács M Németh T Jakus Z Sitaru C Simon E Futosi K . The Src family kinases Hck, Fgr, and Lyn are critical for the generation of the *in vivo* inflammatory environment without a direct role in leukocyte recruitment. J Exp Med. (2014) 211:1993–2011. doi: 10.1084/jem.20132496 PMC4172222 25225462

[21] Káposztás E Balogh L Mócsai A Kemecsei E Jakus Z Németh T . The selective inhibition of the Syk tyrosine kinase ameliorates experimental autoimmune arthritis. Front Immunol. (2023) 14:1279155. doi: 10.3389/fimmu.2023.1279155 PMC10725968 38111569

[22] Jakus Z Németh T Verbeek JS Mócsai A . Critical but overlapping role of FcγRIII and FcγRIV in activation of murine neutrophils by immobilized immune complexes. J Immunol. (2008) 180:618–29. doi: 10.4049/jimmunol.180.1.618 18097064 PMC2647079

[23] Németh T Futosi K Hably C Brouns MR Jakob SM Kovács M . Neutrophil functions and autoimmune arthritis in the absence of p190RhoGAP: generation and analysis of a novel null mutation in mice. J Immunol. (2010) 185:3064–75. doi: 10.4049/jimmunol.0904163 PMC3064944 20675588

[24] Koga H Prost-Squarcioni C Iwata H Jonkman MF Ludwig RJ Bieber K . Epidermolysis bullosa acquisita: the 2019 update. Front Med (Lausanne). (2019) 5:362. doi: 10.3389/fmed.2018.00362 30687710 PMC6335340

[25] Németh T Mócsai A . Feedback amplification of neutrophil function. Trends Immunol. (2016) 37:412–24. doi: 10.1016/j.it.2016.04.002 27157638

[26] Passegué E Wagner EF Weissman IL . JunB deficiency leads to a myeloproliferative disorder arising from hematopoietic stem cells. Cell. (2004) 119:431–43. doi: 10.1016/j.cell.2004.10.010 15507213

[27] McGreal EP Rosas M Brown GD Zamze S Wong SY Gordon S . The carbohydrate-recognition domain of Dectin-2 is a C-type lectin with specificity for high mannose. Glycobiology. (2006) 16:422–30. doi: 10.1093/glycob/cwj077 16423983

[28] Boesch AW Brown EP Cheng HD Ofori MO Normandin E Nigrovic PA . Highly parallel characterization of IgG Fc binding interactions. MAbs. (2014) 6:915–27. doi: 10.4161/mabs.28808 24927273 PMC4171026

[29] Mócsai A Ruland J Tybulewicz VL . The SYK tyrosine kinase: a crucial player in diverse biological functions. Nat Rev Immunol. (2010) 10:387–402. doi: 10.1038/nri2765 PMC4782221 20467426

[30] Németh T Futosi K Szabó M Aradi P Saito T Mócsai A . Importance of Fc receptor γ-chain ITAM tyrosines in neutrophil activation and *in vivo* autoimmune arthritis. Front Immunol. (2019) 10:252. doi: 10.3389/fimmu.2019.00252 PMC6397848 30858848

[31] Jakus Z Simon E Balázs B Mócsai A . Genetic deficiency of Syk protects mice from autoantibody-induced arthritis. Arthritis Rheum. (2010) 62:1899–910. doi: 10.1002/art.27438 20201079 PMC2972644

[32] Kerscher B Willment JA Brown GD . The Dectin-2 family of C-type lectin-like receptors: an update. Int Immunol. (2013) 25:271–7. doi: 10.1093/intimm/dxt006 23606632 PMC3631001

[33] Solomon S Rajasekaran N Jeisy-Walder E Snapper SB Illges H . A crucial role for macrophages in the pathology of K/BxN serum-induced arthritis. Eur J Immunol. (2005) 35:3064–73. doi: 10.1002/eji.200526167 16180250

[34] Akbarzadeh R Czyz C Thomsen SY Schilf P Murthy S Sadik CD . Monocyte populations are involved in the pathogenesis of experimental epidermolysis bullosa acquisita. Front Immunol. (2023) 14:1241461. doi: 10.3389/fimmu.2023.1241461 38116004 PMC10728641

[35] Jonsson H Allen P Peng SL . Inflammatory arthritis requires Foxo3a to prevent Fas ligand-induced neutrophil apoptosis. Nat Med. (2005) 11:666–71. doi: 10.1038/nm1248 15895074

[36] Monach PA Nigrovic PA Chen M Hock H Lee DM Benoist C . Neutrophils in a mouse model of autoantibody-mediated arthritis: critical producers of Fc receptor γ, the receptor for C5a, and lymphocyte function-associated antigen 1. Arthritis Rheum. (2010) 62:753–64. doi: 10.1002/art.27238 20191628 PMC3057458

[37] Haeger SC Kridin K Pieper M Griewahn L Nimmerjahn F Zillikens D . Therapeutic effects of Fc gamma RIV inhibition are mediated by selectively blocking immune complex-induced neutrophil activation in epidermolysis bullosa acquisita. Front Immunol. (2022) 13:938306. doi: 10.3389/fimmu.2022.938306 36311755 PMC9606225

