# Supplementary material for: The C-type lectin receptor Dectin-2 is crucial for the development of experimental autoimmune skin disease
Source: Front Immunol. 2026 Oct 28;17:1962664. doi: 10.3389/fimmu.2026.1962664 (PMC13752007; doi:10.3389/fimmu.2026.1962664)
Supplement: Supplementary file 1 [file Image1.pdf]

Balogh et al.
Supplementary Figure 1

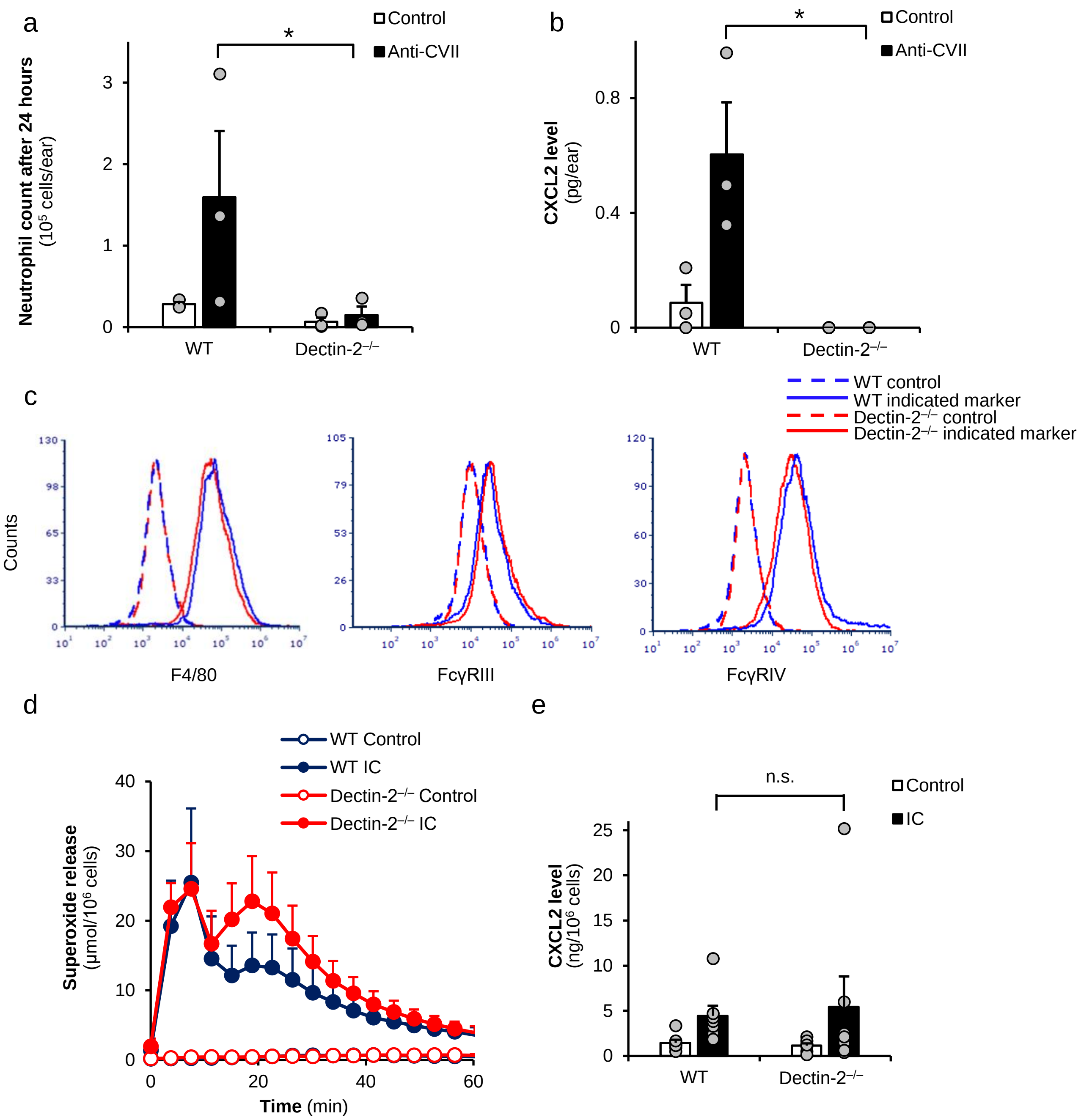

Supplementary Figure 1

Extended data for the modified Arthus reaction in Dectin-2$^{-/-}$ mice and the characterization of Dectin-2$^{-/-}$ macrophages.

(a) In vivo neutrophil recruitment in the ears of WT and Dectin-2$^{-/-}$ mice 24 hours after the induction of the reaction. **(b)** CXCL2 levels were measured in the ears of WT and Dectin-2$^{-/-}$ mice. **(c)** Cell surface molecule expression of WT and Dectin-2$^{-/-}$ BMDMs. **(d-e)** Superoxide and CXCL2 production of HSA-αHSA IC-stimulated WT and Dectin-2$^{-/-}$ BMDMs. Graph in panel **a** shows mean and SEM from 6 mice per genotype from 3 independent experiments. Graph in panel **b** shows mean and SEM from 3 independent experiments. Graph in panels **d-e** show mean and SEM from 7-8 independent experiments. Panels **c** contains representative graphs from 3 independent experiments. See the text for actual P-values. Anti-CVII, anti-type VII collagen antibody; BMDM, bone marrow-derived macrophage; CXCL2, C-X-C motif chemokine ligand 2; IC, immune complex; FcγR, Fcγ receptor; SEM, standard error of the mean; WT, wild type; *$p < 0.05$; n.s., statistically non significant.
